# [β-Glu^2^]TRH Is a Functional Antagonist of Thyrotropin-Releasing Hormone (TRH) in the Rodent Brain

**DOI:** 10.3390/ijms22126230

**Published:** 2021-06-09

**Authors:** Katalin Prokai-Tatrai, Vien Nguyen, Laszlo Prokai

**Affiliations:** Department of Pharmacology and Neuroscience, University of North Texas Health Science Center, Fort Worth, TX 76107, USA; Vien.Nguyen@unthsc.edu

**Keywords:** thyrotropin-releasing hormone (TRH), peptide analogues, [β-Glu^2^]TRH, functional antagonist, analepsia, antidepressant, acetylcholine, in vivo microdialysis

## Abstract

Selective antagonists of thyrotropin-releasing hormone (TRH; pGlu-His-Pro-NH_2_), in order to enable a better understanding of this peptide’s central functions, have not been identified. Using pGlu-Glu-Pro-NH_2_ ([Glu^2^]TRH) as a lead peptide and with modification at its central residue, our studies focused on some of its analogues synthesized as potential functional antagonists of TRH in the rodent brain. Among the peptides studied, the novel isomeric analogue [β-Glu^2^]TRH was found to suppress the analeptic and antidepressant-like pharmacological activities of TRH without eliciting intrinsic effects in these paradigms. [β-Glu^2^]TRH also completely reversed TRH’s stimulation of acetylcholine turnover in the rat hippocampus without a cholinergic activity of its own, which was demonstrated through in vivo microdialysis experiments. Altogether, [β-Glu^2^]TRH emerged as the first selective functional antagonist of TRH’s prominent cholinergic actions, by which this endogenous peptide elicits a vast array of central effects.

## 1. Introduction

**Figure 1:**
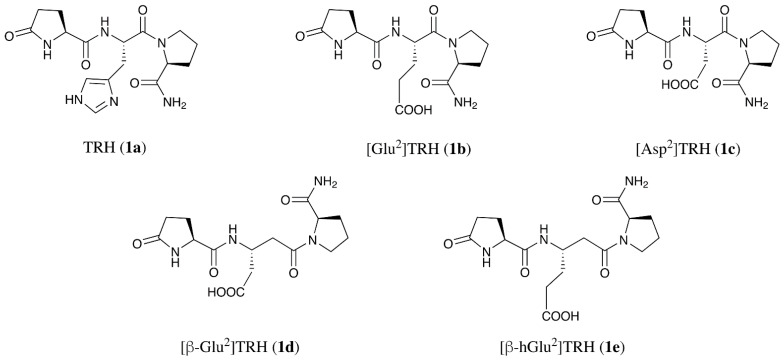
Chemical structure of thyrotropin-releasing hormone (TRH) (**1a**), [Glu^2^TRH (**1b**), and the evaluated [Glu^2^]TRH-like peptides: [Asp^2^]TRH (**1c**), [β-Glu^2^]TRH (**1d**), and [β-hGlu^2^]TRH (**1e**). Structures as shown represent the central residues in an L-configuration.

The thyrotropin-releasing hormone (TRH; pGlu-His-Pro-NH_2_, **1a** in Figure 1) is a hypothalamic hormone that has also been recognized as a neuropeptide with profound impacts on the brain [1,2,3]. This peptide’s central actions are diverse and they affect central nervous system (CNS) chemistry, physiology, and behavior [4,5,6]. Various neurotransmitters have been implicated in these processes, which prominently include the cholinergic system [3,6]. Because of the beneficial central effects, TRH and its analogues have been considered as potential neurotherapeutics [7,8,9,10,11,12,13]. Although two types of G protein-coupled receptors have been identified for TRH in the rodent brain (TRH-R1 and TRH-R2) [14] and another receptor target has also been shown for the peptide [15], only a single receptor similar to the rodent TRH-R1 is expressed in the human brain [16]. Furthermore, it is likely that TRH and most TRH analogues exert their CNS effects through a single receptor [17]. Despite several investigations devoted to the characterization of its receptors [14,16], much remains to be learned about their roles in mediating the CNS functions of TRH. Albeit inverse agonists for studying constitutive signaling activity of TRH receptors have been identified as molecular tools [18], the lack of selective TRH antagonists has been considered an obstacle to a better understanding of this peptide’s central impacts [19] and pharmacological characterizations of TRH analogues with therapeutic promises [12,13].

Several endogenous and synthetic TRH-like peptides with the pGlu-Xaa-Pro-NH_2_ ([Xaa^2^]TRH) sequence have been investigated, where Xaa denotes any natural and unnatural amino acid residues [20,21,22]. For example, [Glu^2^]TRH (**1b**; Figure 1) exhibits various CNS actions characteristic to TRH without endocrine effects, which is preferable in the context of CNS drug development [23]. We have previously shown that, unlike TRH, this peptide did not increase acetylcholine (ACh) turnover in the rat hippocampus [24]. However, **1b** moderately antagonized TRH’s profound cholinergic effect when it was co-perfused with TRH using in vivo microdialysis. Concomitantly, [Glu^2^]TRH (**1b**) at an equimolar dose also partially reversed TRH-evoked analepsia and, thus, it antagonized TRH’s ability to wake up mice from barbiturate-induced narcosis [25], while exhibiting a modest analeptic effect on its own. These observations strongly suggest a modest negative modulatory effect of **1b** on the cholinergic actions of TRH, and also imply the lack of involvement of the cholinergic system in [Glu^2^]TRH’s intrinsic analeptic effect.

Taking [Glu^2^]TRH as a lead compound in the search for functional antagonists of TRH, here, we assessed some of its analogues in which the central Glu was replaced with a structurally similar or an isomeric acidic amino acid residue. We evaluated the resultant tripeptides for their abilities to trigger typical TRH-like central pharmacological actions involving the cholinergic mechanism (such as analeptic and antidepressant-like effects), as well as to counteract these effects elicited by TRH in the brain [24,25]. Structurally related functional antagonists or negative modulators of TRH that do not elicit typical intrinsic central agonist activities of the target peptide in the rodent brain may serve as valuable molecular tools for the elucidation of the cholinergic mechanisms by which TRH exerts its vast array of CNS effects [19].

Accordingly, in this study, we investigated the potential CNS effects of [Glu^2^]TRH-like tripeptides. The replacement of Glu with Asp brought about peptide **1c** (Figure 1) [20]. Analogues **1d** and **1e** with β-Glu and β-homoGlu (β-hGlu) central acidic residue, respectively, were included based on the increased use of β-amino acids because of their unique structural characteristics in the design of the bioactive ligands [26]. These tripeptides (**1c**–**e**) were explored in animal models assessing analeptic and antidepressant-like activities typical for TRH as a neuropeptide [27,28], as well as for their potential to impact these CNS effects of TRH [25]. Selected peptides were also investigated by an in vivo study in order to follow the extracellular ACh turnover in freely moving animals upon perfusing these peptides (alone or together with TRH) through a microdialysis probe placed in the rat hippocampus, and with simultaneous sampling for the determination of changes in ACh levels [24].

## 2. Results and Discussion

### 2.1. Analeptic Effects

**Figure 2:**
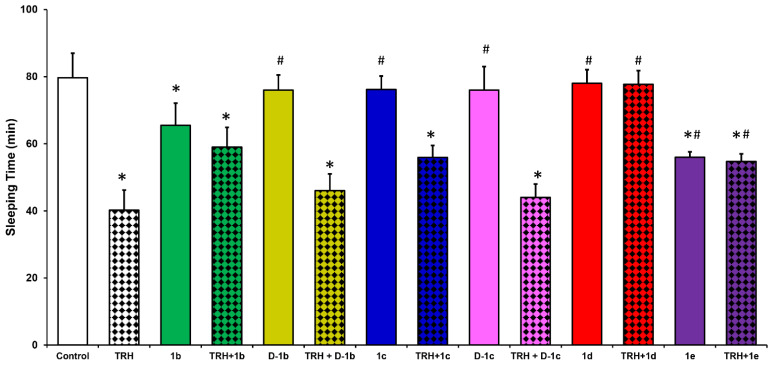
Reversal of pentobarbital-induced narcosis in CD-1 mice by [Glu^2^]TRH-like peptides (Figure 1) alone or when co-administered with TRH. Ten minutes after i.v. injection of test agent alone or together with TRH (10 µmol/kg body weight, each), pentobarbital (60 mg/kg body weight, i.p.) was administered. The sleeping time was recorded from the onset of the loss of righting reflex until the reflex was regained. Data are given as mean ± standard deviation (SD). Analysis of variance (ANOVA) followed by post hoc Tukey’s tests (*p* < 0.05, *n* = 10–14): * indicates statistically significant difference from control, ^#^ indicates statistically significant difference from TRH alone.

As mentioned above, we have previously shown that [Glu^2^]TRH (**1b**) partially reversed TRH’s analeptic effect in mice [25]. In this paradigm, the administration of 10 µmol/kg body weight TRH resulted in 40 ± 5 min sleeping time, while in the saline vehicle-treated group this measure was 80 ± 6 min after the pentobarbital challenge. Compared with TRH, [Glu^2^]TRH (**1b**) produced a modest but statistically significant reduction in sleeping time in relation to the control group (to 66 ± 6 min, Figure 2). Similar to other TRH-like peptides, **1b** has been proposed to act as a weak agonist at TRH receptors [27]. The replacement of Glu with its D-enantiomer rendered the tripeptide **D-1b** inactive (based on the sleeping time of 76 ± 2 min) in this paradigm.

Here, we investigated whether the structurally related tripeptides shown in Figure 1 (**1c**–**e**) would also produce this typical TRH-associated neuropharmacological response [25], as well as their potential to counteract the analeptic effect of TRH. As shown in Figure 2, the intravenous (i.v.) administration of 10 µmol/kg body weight [β-hGlu^2^]TRH (**1e**) produced a statistically significant (*p* < 0.05) analepsia (56 ± 9 min sleeping time) compared with the control group. Therefore, this β-peptide preserved the analeptic activity seen for the corresponding α-peptide (**1b**). Additionally, when co-administered with TRH, the modulation of TRH’s narcosis reduction was also similar to the antagonism recognized for **1b**; i.e., essentially both peptides behaved similarly in this animal model. However, the replacement of Glu with its β-isomer (**1d**) or Asp (**1c**) completely abolished this cholinergic CNS effect of TRH. Accordingly, when animals were treated with [β-Glu^2^]TRH (**1d**) or [Asp^2^]TRH (**1c**) the measured sleeping times (78 ± 12 min and 76 ± 9, respectively) were statistically indistinguishable from that of the control group (80 ± 7 min), as shown in Figure 2.

Similar to what we have seen for [D-Glu^2^]TRH (**D-1b**, 76 ± 4 min sleeping time), D-Asp also rendered the resultant tripeptide (**D-1c**) inert in this study (77 ± 7 min sleeping time). Figure 2 also shows that [β-Glu^2^]TRH (**1d**) completely counteracted TRH’s analeptic effect upon co-administration, as the measured sleeping time (78 ± 12 min) was indistinguishable from that of the control. Dose-response studies also revealed that the median effective dose (ED_50_) for the antagonism was 2.93 ± 2.31 µmol/kg body weight. ED_50_ for the modest antagonistic effect of **1b** was 4.83 ± 0.33 µmol/kg body weight. When [Asp^2^]TRH (**1c**, also without innate analeptic effect) was co-injected with TRH at an equimolar dose (10 µmol/kg body weight), a similar modest decrease in TRH’s analeptic effect was established compared to what we have seen for [β-hGlu^2^]TRH (**1e**); however, the latter exhibited a modest analeptic effect on its own.

Altogether, two [Glu^2^]TRH-like compounds with either Asp or β-Glu central acidic residue (**1c** and **1d**, Figure 1) emerged as non-analeptic tripeptides, but only [β-Glu^2^]TRH (**1d**) was able to completely antagonize this prominent central effect of TRH linked to predominantly cholinergic mechanisms [3,4]. Specifically, the sleeping time (78 ± 12 min) was statistically not different from that of the control (80 ± 7 min) when the mice were injected with an equimolar mixture of TRH and **1d**. Like [Glu^2^]TRH (**1b**), [Asp^2^]TRH (**1c**) only partially antagonized this effect of TRH, as an approximately 40% increase in sleeping time in relation to TRH alone was established upon the co-administration of this peptide with TRH. The replacement of the central residue with the corresponding D-amino acid residue resulted in peptides (**D-1b** and **D-1c**) that were inert in this animal model (Figure 2). This result strongly implied that the observed neuropharmacological action was mediated by a receptor, and that the L-configuration of the central residue is needed for the optimal supramolecular interaction between this set of ligands and their receptor.

### 2.2. Porsolt Swim Tests

**Figure 3:**
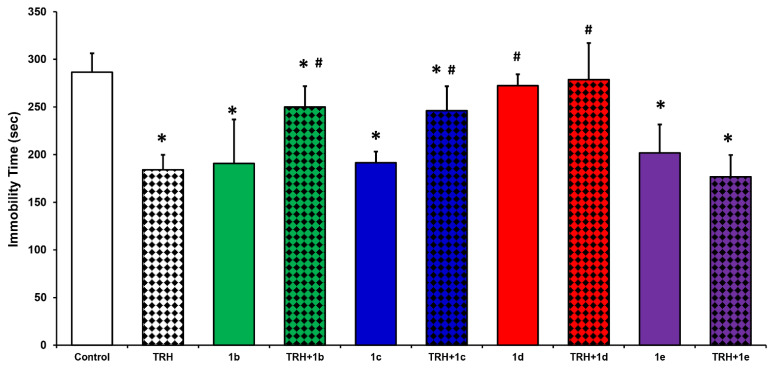
Mean immobility times in the Porsolt swim test for animals injected with [Glu^2^]TRH-like peptides (**1b**–**d**, Figure 1) alone or together with TRH (i.p., 3 µmol/kg body weight, each). The control animals received saline vehicle (30 µL). An equimolar dose of TRH was used as a positive control. The behavioral test began 30 min after the test agent administration. Each experiment was conducted for 6 min, and the duration of immobility was recorded during the last 4 min. Immobility of the animal is defined as floating motionless, making only movements to keep the head above the water. An antidepressant-like effect is indicated by a decrease in the duration of immobility compared with the control group. Data are expressed as average ± SD. ANOVA followed by post hoc Tukey’s tests (*p* < 0.05, *n* = 10–12): * indicates statistically significant difference from control, ^#^ indicates statistically significant difference from TRH alone.

TRH’s antidepressant effect is also well known [1] and it is a frequently used preclinical screening measure in TRH-related drug discovery [28,29,30,31]. One of the most convenient and frequently utilized models for surveying the antidepressant-like effect of potential CNS agents is the Porsolt swim test (PST) [32]. In this paradigm, a reduction of immobility time associated with a depressive state is considered as an antidepressant-like effect. TRH produces an approximately 64% decrease in immobility time in PST compared with that of the saline vehicle control group (287 ± 20 s immobility time) [29,30,31]. At an equimolar dose (3 µmol/kg body weight), [Glu^2^]TRH (**1b**) also significantly decreases the sleeping time (191 ± 26 s) to an extent that is comparable with what we have seen for TRH (Figure 3).

In this study, all of the TRH analogues except [β-Glu^2^]TRH (**1d**) essentially behaved like [Glu^2^]TRH (**1b**). Interestingly, [Asp^2^]TRH (**1c**), which did not produce an appreciable analeptic effect (Figure 2), brought about a similar antidepressant-like response (192 ± 12 s immobility time) as **1b**. The tripeptide with central β-hGlu residue (**1e**) that exhibited some innate analeptic activity as an agonist (Figure 2) also produced an approximately 30% decrease in the immobility time, as shown in Figure 3. The negative modulatory effect of [Asp^2^]TRH (**1c**) on TRH’s antidepressant-like action was also similar to that of [Glu^2^]TRH (**1b**), which brought about an approximately 35% increase in immobility time compared with TRH alone (184 ± 16 s) when the two peptides were co-administered (Figure 3).

In agreement with the analeptic study (Figure 2), [β-Glu^2^]TRH (**1d**) alone also was inert in this paradigm, because it did not exhibit an innate antidepressant-like effect as an agonist (Figure 3). On the other hand, this peptide was able to completely reverse TRH’s antidepressant-like effect, as the measured immobility time (265 ± 32 s) upon the co-administration of the two peptides at equimolar doses (3 µmol/kg body weight) was statistically not different from that of the control group (287 ± 20 s). Altogether, all but [β-Glu^2^]TRH (**1d**) among the peptides shown in Figure 1 exhibited modest innate agonist activities in the PST model under the experimental conditions used. When co-administered with TRH, [β-hGlu^2^]TRH (**1e**) had no impact, while [Glu^2^]TRH and [Asp^2^]TRH (**1b** and **1c**, respectively) both showed modest functional antagonism, as summarized in Figure 3. Importantly, as in the analeptic study, [β-Glu^2^]TRH (**1d**) had the ability to antagonize the antidepressant-like effect of TRH without exhibiting this CNS activity on its own.

### 2.3. Hippocampal ACh Turnover

TRH exerts its numerous CNS actions most stoutly in the hippocampus through the involvement of cholinergic neurons [3,4]. These actions include the above monitored analeptic and antidepressant-like effects of the peptide [3,4,19]. A plausible confirmation of the functional involvement of central cholinergic neurons in these effects can be achieved through in vivo microdialysis samplings of the extracellular fluid from the target brain region in freely moving animals and, then, measuring the endogenous ACh contents in the collected perfusates [24,31,33]. This neurochemical assessment was expected to be complementary to the neuropharmacological studies shown in Figure 2 and Figure 3.

As established before, TRH produces an approximately 3.5-fold increase in this neurotransmitter level, while [Glu^2^]TRH (**1b**) does not influence ACh turnover [24,31]. In our studies, the equilibrium baseline ACh level reached before the start of a test agent’s perfusion was taken as 100%. ACh levels obtained at steady state (usually after 1 h) after the perfusion of the test agent alone or together with TRH are expressed as percent changes compared with the equilibrium baseline level representing the perfusion of neostigmine-containing artificial cerebrospinal fluid (aCSF) only [24].

**Figure 4:**
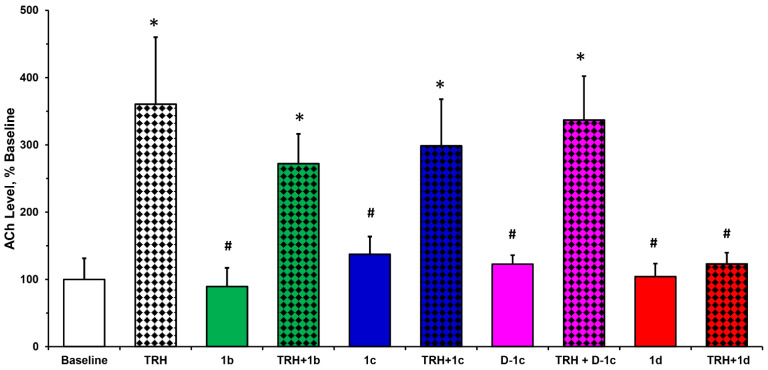
Percent changes in hippocampal ACh levels upon perfusing test agents alone or together with an equimolar concentration of TRH (3 µM, each in aCSF containing 2 µM neostigmine). The steady-state baseline ACh level was measured in the microdialysates collected for 2 h in 20-min fractions after 1 h of the initiation of the experiment. Then, perfusion was switched to a solution containing TRH and/or the test agent dissolved in aCSF containing 2 µM neostigmine. ACh levels after the test agents’ perfusions were expressed as percent changes in 3–6 fractions after equilibration compared with the steady state baseline ACh levels taken as 100%. ANOVA followed by post hoc Tukey’s tests (*p* < 0.05, *n* = 4): * indicates statistically significant difference from baseline control, ^#^ indicates statistically significant difference from TRH alone.

Figure 4 shows that, like [Glu^2^]TRH (**1b**), none of the test agents (**1c**–**1e**) exhibited innate effects on the hippocampal ACh turnover. The peptide with Asp central residue (**1c**) produced a modest antagonism against TRH’s increase of ACh production (298 ± 70% ACh level in relation to 100 ± 31% baseline), similar to that of **1b**. The corresponding D-isomer (**D-1c**) was also ineffective alone (122 ± 13% ACh level compared with baseline), or when perfused together with TRH (337 ± 65% ACh level compared with baseline), in agreement with the pharmacological assessments shown in Figure 2 and Figure 3.

[β-Glu^2^]TRH (**1d**), on the other hand, completely reversed the endogenous ACh release elicited by TRH and, therefore, the measured neurotransmitter level increase (123 ± 27%) was statistically not different from that of the equilibrium baseline (100 ± 31%). This observation confirms those seen in the analeptic (Figure 2) and PST (Figure 3) models, where **1d** completely reversed these typical CNS effects of TRH; thus, upon co-administration with TRH, the measured values were statistically indistinguishable from those of the control groups receiving the saline vehicle only. Accordingly, TRH administration indeed increased the ACh turnover in the hippocampus of pentobarbital-anaesthetized mice (Figure 2) or when the animals were exposed to stress conditions seen in PST (Figure 3). In turn, this cholinergic activity could be antagonized selectively by [β-Glu^2^]TRH (**1d**). Therefore, based on the outcomes of our studies reported here, we propose that the novel [β-Glu^2^]TRH (**1d**, Figure 1) is the first functional antagonist of the central cholinergic mechanism prominently exhibited through various CNS actions by TRH [2,14].

## 3. Materials and Methods

### 3.1. Materials

All of the chemicals used were reagent or peptide synthesis grade. Benzotriazole-1-yl-oxy-tris-pyrrolidino-phosphonium hexafluorophosphate (PyBOP), 1-hydroxybenzo-triazole (HOBt), *N*,*N*’-diisopropyl-ethylamine (DIPEA), and trifluoroacetic acid (TFA) were obtained from MilliporeSigma (St. Louis, MO, USA). Fmoc-Rink Amide resin (Fmoc denotes fluorenylmethyloxycarbonyl), amino acids, TRH, and [Glu^2^]TRH were purchased from Bachem Americas, Inc. (Torrance, CA, USA). Artificial cerebrospinal fluid (aCSF) and neostigmine bromide were supplied by Harvard Apparatus (Holliston, MA, USA) and MilliporeSigma, respectively. The solvents were purchased from Thermo Fisher Scientific (Waltham, MA, USA).

### 3.2. Animals

Male rodents were used in these studies; specifically, CD-1 mice (40 ± 10 g) and Sprague-Dawley rats (275 ± 6 g body weight), which were purchased from Charles River Laboratories (Wilmington, MA, USA). The animals were housed as two rats/cage and five mice/cage under common 12-h/12-h light/dark cycle, with free access to food and water. All of the procedures were reviewed and approved by the institutional animal care and use committee (IACUC) before the initiation of the studies. For the neuropharmacological and neurochemical assessments, each animal was used only once. Altogether, 326 mice and 36 rats were used in our studies.

### 3.3. Instruments

For purification of the synthesized peptides, preparative reversed-phase high-performance liquid chromatography (RP-HPLC) was done using a system consisting of a Thermo Separations (Fremont, CA, USA) SpectraSERIES P200 binary gradient pump, a Rheodyne (Cotati, CA, USA) model 7125 injector valve equipped with a 5 mL loop, and a Spectra 100 UV/vis detector set at detection wavelength of 216 nm [23,29]. Analytical RP-HPLC was performed using an Agilent (Palo Alto, CA, USA) 1050 system with UV detection at 220 nm. The semi-preparative RP column was Waters (Milford, MA, USA) RCM DeltaPack C18, 250 mm × 100 mm i.d., and the analytical column was Thermo Hypersil-Keystone Betabasic-8 (100 mm × 4.6 mm i.d., 5 µm particles) operated at 10 mL/min and 1.0 mL/min flow rates, respectively. The mobile phases were mixed from 0.1% (*v*/*v*) TFA in H_2_O and 0.08% (*v*/*v*) of TFA in acetonitrile. Electrospray ionization (ESI) mass spectra (MS) were obtained using a quadrupole ion trap instrument (LCQ, Thermo Finnigan, San Jose, CA, USA).

### 3.4. Synthesis

Manual solid-phase peptide synthesis was done as reported before [23,29,31] on 1 g of pre-loaded (Fmoc)-Pro-Rink-MBHA-Amide resin (0.48 meq/g), utilizing standard Fmoc-chemistry with PyBOP coupling in the presence of HOBt/DIPEA and using amino acid:PyBOP:DIPEA:HOBt at a 1:1:2:1 molar ratio in *N*,*N*-dimethylformamide. The side-chain carboxylic acid was protected as tert-butyl ester. The tripeptides were cleaved from the solid support with a mixture of TFA:H_2_O:(i-Pr)_3_SiH (95:2.5:2.5, *v*/*v*/*v*, 10 mL, 1 h). After lyophilization, the crude peptides were purified by preparative RP-HPLC. Combustion analyses were performed by Atlantic Microlabs Inc. (Atlanta, GA, USA).

[Asp^2^]TRH (**1c**): MS (ESI) *m*/*z* 340; Combustion analysis for C_14_H_20_N_4_O_6_ × 2 H_2_O: Calc.: C, 44.68, H, 6.43; N, 14.89. Found C, 44.74; H, 6.14; N, 14.90.
[D-Asp^2^]TRH (**D-1c**): MS (ESI) *m*/*z* 340; combustion analysis for C_14_H_20_N_4_O_6_ × 2 H_2_O: C, 44.68, H, 6.43; N, 14.89. Found C, 44.96; H, 6.84; N, 14.61.
[β-Glu^2^]TRH (**1d**): MS (ESI) *m*/*z* 354; Combustion analysis for C_15_H_22_N_4_O_6_ × 0.5 H_2_O, Calc.: C, 49.58, H, 6.38; N, 15.42. Found C, 49.53; H, 6.26; N, 15.58.
[β-hGlu^2^]TRH (**1e**): MS (ESI) *m*/*z* 368; Combustion analysis for C_16_H_24_N_4_O_6_ × H_2_O × 0.5 TFA, Calc.: C, 46.05, H, 6.02; N, 12.64. Found C, 46.13; H, 5.73; N, 12.36.

### 3.5. Assessment of Analeptic Activity

These behavioral studies were conducted according to our established procedure [23,25,29]. The test compounds were dissolved in saline and were administered i.v. through the tail vein of the mouse. In the control group, the vehicle alone (30 µL) was injected, while in the treatment groups, equimolar doses of test compounds (10 μmol/kg body weight) were administered 10 min before the pentobarbital challenge. The animals received sodium pentobarbital by intraperitoneal (i.p.) injection at a dose of 60 mg/kg body weight. The sleeping time was recorded by a trained individual unaware of the treatment regimen. Sleeping time is defined as the onset of loss of the righting reflex until the reflex is regained. Data were expressed as mean ± SD for *n* = 10–14 animals per treatment group. In the dose-response studies, six different doses (1, 2, 5 10, 15, and 25 μmol/kg body weight, *n* = 4 mice per group) were used to determine the ED_50_ value of the antagonism.

### 3.6. Assessment of Antidepressent-Like Activity

After the i.p. administration of test agents alone or together with TRH at equimolar concentrations (3 µmol/kg body weight), our validated and previously published PST protocol was used [23,29,31]. Ten to twelve mice per treatment group were tested. The behavior tests began 30 min after the test agent administration. The control group received 30 µL of saline vehicle only. Each experiment was conducted for 6 min, and the immobility time was recorded in the last 4 min. Immobility time is defined as the duration of floating motionless, only making movements necessary to keep the head above the water. Data were expressed as mean ± SD for *n* = 10–14 animals per treatment group.

### 3.7. Measurement of Hippocampal ACh Levels

To assess the modulation of ACh by the test agents or establishing their negative modulatory effect on TRH-induced ACh turnover, microdialysis samplings of the extracellular space of the rat hippocampus were done according to our previously published protocol [24]. The microdialysis experiments were conducted using freely moving animals and a Raturn movement response caging system (Bioanalytical Systems, Inc., West Lafayette, IN, USA). Briefly, a cerebral guide cannula (CMA/Microdialysis, Inc.; Acton, MA, USA) was inserted into the hippocampus before the actual microdialysis probe (CMA/12, CMA/Microdialysis, Inc., with a 4-mm long polycarbonate membrane having 2-kDa molecular weight cut-off) was inserted approximately 7 days after the placement of the guide cannula. The perfusion of the probe at a 2 µL/min flow rate was done with artificial cerebral spinal fluid (aCSF) containing 2 µM neostigmine. For measuring the steady-state baseline ACh level, microdialysates were collected for 2 h in 20-min fractions after 1 h of the initiation of the experiment. Then, perfusion was switched to a solution containing TRH and/or test agents in the medium used for collecting baseline fractions. Microdialysates were collected in a refrigerated fraction collector (Bioanalytical Systems, Inc.) followed by their HPLC assay using amperometric detection [24]. The ACh levels after the test agents’ perfusions were expressed as percent changes in 3–6 fractions after equilibration (usually after 1 h of perfusion), compared with the steady state baseline ACh level taken as 100%.

### 3.8. Data Analysis

Statistical evaluations were done using ANOVA. Two-group comparisons employed post hoc Tukey’s test, and *p* < 0.05 was considered statistically significant. ED_50_ values were calculated using the Scientist software (MicroMath, St. Louis, MO, USA) by fitting the results of the dose-response experiments to the equation ∆ = ∆_max_/[1 + (ED_50_/D_i_)^h^], where ∆ and ∆_max_ are the average decrease and maximal measured average decrease in sleeping time (min) compared with the control, respectively; D_i_ is the dose (µmol/kg body weight); and h is the Hill coefficient [25,34].

## 4. Conclusions

Using [Glu^2^]TRH (**1b**) as a lead peptide, our pharmacological and neurochemical studies focusing on some of its analogues with a structurally similar or an isomeric central acidic residue (**1c**–**1e**) have identified [β-Glu^2^]TRH (**1d**) as the first selective functional antagonist of the cholinergic action prominently elicited by TRH (**1a**) in the rodent brain. As the lack of selective TRH antagonists has been considered to be an obstacle not only for a better understanding of this peptide’s central impacts, but also for the pharmacological characterizations of TRH analogues with therapeutic promises, further investigations are highly justified regarding the molecular basis of **1d**’s action on TRH in the CNS.

## Data Availability

All data sets are available upon reasonable request from the corresponding authors.
